# Modified screening method of middle american dry bean genotypes reveals new genomic regions on Pv10 associated with anthracnose resistance

**DOI:** 10.3389/fpls.2022.1015583

**Published:** 2022-11-15

**Authors:** Kristin J. Simons, Stephan Schröder, Atena Oladzad, Phillip E. McClean, Robert L. Conner, Waldo C. Penner, Dennis B. Stoesz, Juan M. Osorno

**Affiliations:** ^1^ Department of Plant Sciences, North Dakota State University, Fargo, ND, United States; ^2^ Morden Research and Development Centre, Agriculture and Agri-Food Canada, Morden, MB, Canada

**Keywords:** anthracnose, dry bean, MDP, fungal resistance, GWAS, ARR

## Abstract

Anthracnose, caused by the fungal pathogen *Colletotrichum lindemuthianum* (Sacc. & Magnus) Lams.-Scrib., is one of the most devastating diseases in dry bean (*Phaseolus vulgaris* L.) with seed yield losses up to 100%. Most anthracnose resistance genes thus far identified behave in a dominant manner and were identified by seedling screening. The Middle American Diversity Panel (MDP; n=266) was screened with a modified greenhouse screening method to evaluate the response to anthracnose race 73. Thirty MDP genotypes exhibited resistance to the race of which 16 genotypes were not known to contain anthracnose resistance genes to race 73. GWAS with ~93,000 SNP markers identified four genomic regions, two each on Pv01 and Pv10, associated race 73 resistance. A likelihood-ratio-based R2 analysis indicated the peak four SNP markers are responsible for 26% of the observed phenotypic variation, where one SNP, S10_072250, explains 23% of the total variation. SNP S10_072250 is associated with a new region of anthracnose resistance and is in an intron of a *ZPR1*-like gene. Further greenhouse testing of the 16 resistant lines without previously known resistance to race 73 revealed various levels of resistance under various levels of disease pressure. Disease resistance was further characterized in the field using four representative genotypes. GTS-900 and Remington exhibited field resistance while Merlot and Maverick were susceptible. Field testing with two different fungicide regimes revealed the resistant genotypes had no significant disease differences. The results suggest resistance to anthracnose may differ at various growth stages and that breeders have been selecting for major genes at early seedling stages while ignoring the effect of alternative genes that may be active at later stages. The newly identified resistant lines may be related to Age Related Resistance (ARR) and could be exploited as parental sources of anthracnose resistance in addition to already known major genes. The physical localization of the multiple regions of resistance confirms the presence of two clusters of disease resistance genes on Pv01 and identifies two new regions of anthracnose resistance on Pv10 possibly associated with ARR. Future research should look at the mode of inheritance of this resistance and its effect when combined with other anthracnose resistance loci.

## Introduction

*Colletotrichum lindemuthianum* (Sacc. & Magnus) Lams.-Scrib. is the causal agent of a devastating fungal disease of dry beans known as anthracnose, which occurs in almost all bean growing regions worldwide (Sharma et al., 2007). This results in a decreased seed quality and can also cause seed yield losses up to 100% if disease onset occurs at early stages (Junaid et al., 2014). The world produced 27 million MT of dry beans in 2020, worth an estimated $30 billion (US) of which dry beans in the Americas contributed 30% of the total (https://www.fao.org/faostat). United States is one of the largest dry bean producers in the world, with a total annual production of 1.6 million MT, and a production value of close to USD $1 billion in 2020 (USDA-NASS, 2020). The most prevalent race in the United States is race 73, to which most Middle American cultivars from the market classes commonly grown in the US (i.e., pinto, navy, black, great northern and small red) are susceptible (Balardin et al., 1997). However, disease response varies within each market class depending on the genetic background of each genotype. Increasing the number of resistant genotypes will decrease overall economic losses and provide higher seed quality.

Twenty-four resistance loci have been identified on eight chromosomes, Pv01, Pv02, Pv03, Pv04, Pv07, Pv08, Pv09 and Pv11, in various dry bean cultivars and germplasm (González et al., 2015; Zuiderveen et al., 2016). The mode of inheritance of most anthracnose resistance genes follows a typical dominant, single gene model. One of the most effective pyramiding of anthracnose resistance genes involves the *Co-1^2^* and *Co-4^2^* alleles, which are on Pv01 and Pv08, respectively. In addition to the *Co*-genes, the physiological state and the age of the bean plant may contribute to anthracnose resistance in an age-related resistance (ARR) manner. According to Develey-Rivière and Galiana (2007), the mechanisms behind ARR in plants vary, are species-specific, and can be due to specific gene regulations/expression levels in different plant tissues and development stages. Thus far, increased levels of proteins related to resistance, such as phenylalanine ammonia-lyase (*PAL*), chalcone synthase (*CHS*), and chalcone isomerase (*CHI*), which accumulate in the plant tissue over time, have been reported in some species (Nuss et al., 1996).

A classic example of ARR was reported in the cereal rust patho-system. Two well studied genes for ARR, classified as adult plant resistance genes, include the genes, *Lr34* and *Sr2* (reviewed in Ellis et al., 2014). These two cereal rust resistant genes provide partial, broad-spectrum resistance. *Sr2*, a resistance gene for stem rust (*Puccinia graminis* sp. *Tritici* Eriksson & Henning), provides both partial resistance as well as a boost for other R genes. *Lr34* provides partial resistance against leaf rust (caused by *P. triticina* Eriksson), stripe rust (*P. striiformis* Westendorp), and powdery mildew (*Blumeria graminis* (de Candolle) Speer). It was cloned in 2009 (Krattinger et al, 2009) and is related to the ABC class of trans-membrane transporters. Both *Sr2* and *Lr34* have been extensively used with R genes in breeding programs to pyramid resistance.

ARR has been extensively reported in various plant species including dry beans. Bateman and Lumsden (1965) reported an increase of resistance to *Rhizoctonia solani* Kuehn (isolate R-B) in the hypocotyls of red kidney beans two weeks after emergence, and full resistance after four weeks. The conversion of pectin to calcium pectate caused increased resistance, due to the inability of *R. solani* (isolate R-B) to hydrolyze calcium pectate and subsequently destroy the cell wall. Beebe and Pastor-Corrales (1991), reported similar observations in seedling assays of the Andean cultivar ICA-Llanogrande, a cargamanto (large cream/mottled seed) climbing bean (growth type IV) cultivar from Colombia, and Rio Negro, a black bean cultivar from Brazil (Voysest, 2000), in which both cultivars were susceptible to anthracnose at the seedling stage, while older plants appeared to be resistant. Another study by Bigirimana and Höfte (2001) detected increased resistance to *C. lindemuthianum* races 385 and 401 over time in the cultivar Prelude (a snap bean cultivar), where 6 to 8-day old plants showed moderate or no resistance, but developed full resistance 12 days after emergence. The authors speculated that similar resistance mechanisms are involved as described in Mezzola et al. (1994), for resistance to bacterial blight (*Xanthomonas oryzae* pv. *oryzae* (Ishiyama) Swings, van den Mooter, Vauterin, Hoste, Gillis, Mew & Kersters) on rice (*Oryza sativa* L.) which was mediated by gene *Xa21*, and was not expressed at early development stages. In addition to *Xa21* related mechanisms, Bigirimana and Höfte (2001) speculated that developmentally regulated cell death genes may have a role in ARR. However, none of those studies looked further into the plant defense mechanisms at the physiological/biochemical level.

Anthracnose resistance studies in dry beans are usually carried out using young plants (8 days; Castellanos et al., 2015) at the unifoliate leaf stage. This allows for rapid screening of high numbers of genotypes. Our objective was to identify resistance in older plants (similar to ARR), that could be used in dry bean breeding programs. To evaluate the plant defense system in older dry beans, and unveil potentially new and unutilized defense mechanisms, the plants used for this study were substantially older (approx. 14-20 days, trifoliate leaf stage) than in the original method of anthracnose inoculation by Castellanos et al. (2015). The genetic location of the associated resistance genes was identified using the Middle American Diversity Panel (MDP), and the observed resistance was further characterized by additional greenhouse studies with varying lengths of optimal disease conditions, and field testing to confirm the field applicability of the resistance.

## Materials and methods

### Disease evaluation of the middle American diversity panel

The MDP consisted of a collection of 280 dry bean genotypes belonging to the Middle American gene pool, composed of the races Durango/Jalisco (i.e., pinto, great northern, medium red, and pink) and Mesoamerica (i.e., navy and black) (Moghaddam et al., 2016). Approximately 80% of the MDP genotypes were cultivars while the remainder genotypes were improved germplasm and landraces, all considered homozygous lines. Three replicates over time of five plants from each of the 266 MDP genotypes were grown in the greenhouse located in Fargo, ND at 22°C with additional light (600W high pressure sodium lamps) on a 14/10 h day/night cycle. Plants were evaluated as a randomized complete block design (RCBD) and were inoculated at the first trifoliate leaf stage (V2; Van Schoonhoven and Pastor-Corrales, 1987), 14 to 20 days after planting (DAP) with a solution of 1.2x10^6^ conidia/ml of *C. lindemuthianum* race 73, using a mist sprayer until run-off. The genotypes USPT-ANT-1 (*Co-4^2^*) (Miklas et al., 2003) and Envoy (*Co-1^2^* and *Co-4^2^*) (Dongfang et al, 2008) were used as resistant controls to anthracnose race 73. The cultivars Eclipse (Osorno et al., 2009) and Stampede (Osorno et al., 2010) were used as susceptible controls. The inoculated plants were incubated in humidity chambers for five days at 98% to 100% relative humidity under a 12h day/night cycle. Disease severity was evaluated 12 days post inoculation (DPI). For rapid screening (phenotyping) of this initial large number of genotypes, a simple disease severity scale was used, where 1 = no symptoms, 2 = small to enlarged lesions but healthy new tissue growth observed, unifoliate leaves have dropped due to infection; and 3 = numerous enlarged lesions or sunken cankers on the lower sides of leaves or stems, no healthy new tissue/plant death. Disease rating was based on the overall appearance of the plant, the condition of the unifoliate and trifoliate leaves, and new plant growth. This condensed disease rating scale focused on the plant’s ability to stop disease progression and survive, more clearly delineating the susceptible genotypes from the resistant genotypes. The disease reaction was further characterized in a subset of resistant lines using a traditional 1-9 scale as described later. To eliminate outliers, median scores for each genotype were used for the GWAS. Phenotypic data from lines with less than seven phenotypic data points was discarded.

### SNP dataset

A SNP dataset generated from the alignment of MDP sequences to the Middle American reference genotype, UI111, was available from the BeanCAP website (HapMap_469-Pinto UI111-geno-GATK-cleaned-imputed-sorted-hpmp-hmp, version: 1.0, http://arsftfbean.uprm.edu/beancap/research/). The SNP dataset with reference UI111 was chosen since it belongs to the Middle American gene pool versus the G19833 reference which is part of the Andean gene pool (Schmutz et al., 2014). The SNP dataset was generated from genotype-by-sequencing (GBS) reads of 469 MDP genotypes (Oladzad et al., 2019a). The reads were aligned to UI111 v1.0 (https://phytozome-next.jgi.doe.gov/) and variants were called using the GATK Unified Genotyper v3.3 (McKenna A. et al, 2010). SNPs with less than 50% missing data were imputed using the likelihood-based method implemented in fastPHASE 1.3 (Scheet & Stephens, 2006) with default settings and filtered to remove SNP with a MAF below 0.05. The final SNP dataset after filtering for MAF consisted of ~90,000 SNPs specific to the Middle American gene pool and was subsequently used for the association analysis.

### Genome wide association studies

The GWAS was carried out using a generalized linear mixed model within Genome-wide Efficient Mixed Model Association (GEMMA) since the underlying distribution was not normal (Zhou and Stephens, 2012 and Zhou and Stephens, 2014). Phenotypic medians were used in the GWAS to eliminate the influence of outliers. The relationship matrix was calculated using the centered relatedness algorithm within GEMMA (Zhou and Stephens, 2012 and Zhou and Stephens, 2014). One principal component explained 27.5% of the variation and was included in the GWAS model. The mhtplot() function in the R package gap was used to generate the final Manhattan plot (R Core Team, 2013; www.R-project.org). Highly significant markers were defined as those falling outside the 0.01 percentile tail of the empirical distribution of p-values after 10,000 bootstraps (Mamidi et al., 2014; Moghaddam et al., 2016; Oladzad et al., 2019a; Oladzad et al., 2019b; Oladzad et al., 2020; Simons et al., 2021) and drawn as horizontal bars on the Manhattan plots. The phenotypic contribution of the markers was evaluated, using likelihood ratio R2 analyses of peak SNP markers with low p-values using the R package, genABEL (Sun et al., 2010). Candidate genes were selected based on function and location within a ±50kb window of the significant SNP or interval. An unpaired student’s t-test was used to determine if the mean between allelic states for peak SNP within each significant interval were significantly different.

### Plant exposure to a favorable disease environment for an extended period of time

Based on the results of the anthracnose screening of the 266 genotypes of the MDP, 16 genotypes exhibiting resistance to race 73, but with no previous knowledge/report of any anthracnose resistance genes (**Table 1**), were selected for further evaluation to characterize the nature of their anthracnose resistance. The plants were evaluated in RCBD consisting of three replicates, using the same resistant and susceptible checks mentioned previously. Each replicate consisted of five plants for each genotype and each replicate was grown under greenhouse conditions as described above. Five disease exposure times were tested in five separate experiments: Plants at the unifoliate leaf stage (eight DAP; stage V2) were inoculated as described above. The inoculated plants were incubated in humidity chambers for three days at 98% to 100% relative humidity under a 12h day/night cycle (experiment 1). Plants at the first trifoliate leaf stage (14 to 20 DAP to detect potential ARR; stage V3; Van Schoonhoven and Pastor-Corrales, 1987) were also inoculated as described above and incubated in humidity chambers for five days (experiment 2), seven days (experiment 3), nine days (experiment 4) and twelve days (experiment 5), respectively, at 98% to 100% relative humidity under a 12h day/night cycle. Each experiment was performed three times.

**Table 1:** Characteristics and history of MDP genotypes exhibiting resistance under the trifoliate screening method (condensed 1-3 rating scale) including their previously reported anthracnose resistance genes to any race of anthracnose.

Genotype	Market Class	Reported Resistance Gene	Resistance gene reported by	Released by
Loreto	Black	*Co-1*	Kelly et al., 2015	Provita/ADM Seedwest
Raven	Black	*Co-1*	Kelly et al., 1994	Michigan State University
UI-911	Black	unknown	anthracnose resistance not tested (Myers et al., 1997)	University of Idaho
Phantom	Black	*Co-1* and *Co-2*	Kelly et al., 2000	Michigan State University
Jaguar	Black	*Co-1*	Kelly et al., 2001	Michigan State University
Condor	Black	*Co-1*	Kelly et al, 2005	Michigan State University
CDC Nordic	Great Northern	*Co-1^5^*	Dongfang et al., 2008	University of Saskatchewan
Envoy	Navy	*Co-12, Co-4^2^, Co-2?*	Dongfang et al., 2008	Gen-Tec Seeds, Limited
Mackinac	Navy	*Co-1*	Kelly et al., 1998	Michigan State University
N05324	Navy	*Co-1*	Kelly et al., 1998	Michigan State University
Newport	Navy	*Co-1* and *Co-2*	Kelly et al., 1995	Michigan State University
Sanilac	Navy	*Co-1*	Schwarz et al, 1982	Michigan State University
Morden 003	Navy	*Co-5*	Dongfang et al., 2008	Agri Food Canada
Huron	Navy	*Co-1*		Michigan State University
I9365-25	Pink	unknown	*Phaseolus coccineus* L. in pedigree as potential anthracnose resistance (Singh et al., 2014)	USDA Prosser, WA
Grand Mesa	Pinto	unknown	no anthracnose resistance reported (Brick et al., 2005)	Colorado State University
Agassiz	Pinto	unknown		Rogers Seed/ADM
CDC Pintium	Pinto	unknown		University of Saskatchewan
CDC WM-2	Pinto	unknown	partial anthracnose resistance to race 73 reported (Bett et al., 2014)	University of Saskatchewan
GTS-900	Pinto	unknown	resistant to race alpha, beta, gamma, delta (Gen-tec Seed, Limited 2002)	Gen-Tec Seeds, Limited
Maverick	Pinto	unknown		North Dakota State University
USPT-ANT-1	Pinto	*Co-4^2^*	Miklas et al., 2003	USDA Prosser, WA
Win Mor	Pinto	*Co-1?*	Balasubramanian et al., 2008	Agri Food Canada
Remington	Pinto	unknown	anthracnose resistant to race 73	Rogers Seed/ADM
Topaz	Pinto	unknown	anthracnose resistant to race 73	Rogers Seed/ADM
F07-014-22-2	Red	unknown	Compuesto Chimaltenango-2 (Singh and Schwartz, 2010) and Negro Tacana (López-Salinas et al., 1997) in pedigree. Both report anthracnose resistance (race unknown)	Porch/Urrea Shuttle Breeding PR-NE
F07-449-9-3	Red	unknown	Compuesto Chimaltenango-2 (Singh and Schwartz, 2010) and Negro Tacana (López-Salinas et al., 1997) in pedigree. Both report anthracnose resistance (race unknown)	Porch/Urrea Shuttle Breeding PR-NE
Merlot	Small Red	unknown	reported to be susceptible to anthracnose (Hosfield et al., 2004)	Michigan State University
Deorho	Small Red	unknown	partial anthracnose resistance reported - race unknown (Rosas & Escoto, 2007)	Zamorano Univ.
CENTA Pipil	Small Red	unknown	partial anthracnose resistance reported - race unknown (Cabrera & Reyes Castillo, 2008)	Zamorano Univ.

Disease evaluation took place seven days after removal of the plants from the humidity chambers to allow for anthracnose development. To make the results comparable to other studies, and to obtain a more detailed picture about the level of resistance, the standard 1-9 visual scale (Castellanos et al., 2015) was applied instead of the condensed 1-3 scale used to screen the 266 MDP genotypes. The data analysis for this paper was generated using SAS software, Version 9.4 of the SAS System for Windows. Copyright © 2016 SAS Institute Inc. SAS and all other SAS Institute Inc. product or service names are registered trademarks or trademarks of SAS Institute Inc., Cary, NC, USA. The LSMeans and ANOVA were calculated using the GLM procedure. Significant differences between genotypes were identified using the least significant difference (LSD) multiple comparison test.

### Field testing of representative resistant genotypes

The field trials were an international collaboration with plots located at the Morden Research and Development Center at Morden, Canada (49.19, -98.08, 296masl) during the 2017, 2018, and 2019 growing season. The Morden Research and Development Center was an exceptional location as they had previously completed field trials using anthracnose race 73 and thus had natural sources of race 73 inoculum as well as an established field misting system. The trial involved four cultivars representing the 16 newly identified resistant cultivars, GTS-900, Maverick, Merlot, Remington, as well as two checks, Stampede (susceptible check) and USPT-ANT-1 (resistant check). The experiment was arranged in a split block design replicated three times with two inoculation treatments (no inoculation and spore inoculation). The plots consisted of 4 rows, 5 meters in length, 0.3 meters row spacing and 0.6 meters between plots. Plots of soybeans (*Glycine max* L.) with the same dimensions were planted around the bean plots in a checkered pattern to reduce the spread of anthracnose among the plots. Emergence was determined by counting all the seedlings in each row four weeks after planting. The plots in all the spore inoculated treatments were sprayed approximately five weeks after planting. The spore suspension consisting of 1.2 x 10^6^ spores/mL of race 73 of *C. lindemuthianum*, which was applied using a volume of 170 ml per plot. A mist irrigation system was used to promote anthracnose development when rainfall was lacking. The average temperature and rainfall total during the growing season (June 1 to August 31) was 19°C and 14cm, respectively, in 2017, 20°C and 17cm in 2018 and 19°C and 19cm in 2019.

Foliar disease severity in the canopy was rated on ten plants in each plot approximately six weeks after inoculation. Similarly pod infection was rated approximately ten weeks after inoculation at ten sites within each plot. Anthracnose leaf infection was rated on a 1-9 scale (Castellanos et al., 2015) with all leaves on the plant collectively evaluated and pod infection was rated on a percent area infected of all visible pods at each site within the plot. The mean rating of each plot was used for statistical analysis. All plots were harvested and the seed weighed to determine plot yield. A subsample of 200 seeds was collected for each plot and weighed to determine one hundred seed weight. In addition, the number of seeds with anthracnose lesions was counted for each plot. The field data was analyzed by proc GLM using a fixed effect model. Levene’s test for homogeneity of variance was used to determine if the multiple year data could be combined for analysis. Data was not homogeneous across years and thus, each year underwent independent analysis. Significant differences between treatments were tested using the least significant difference (LSD) multiple comparisons test.

### Field testing of fungicide application regimes

With the objective to identify if the presence of ARR in a cultivar could reduce the recommended fungicide application regime to control anthracnose, a second field trial was conducted again at the Morden Research and Development Center and used the same six cultivars during the 2017, 2018, and 2019 growing seasons. The trial was replicated three times in a split block design with block one having one fungicide application and the other block two having three fungicide applications. The foliar applications of the fungicide Headline (pyraclostrobin 250 g/L; BASF, Florham Park, New Jersey) were made at a rate of 385 ml/ha. Block one had one application of Headline at mid bloom. Block two had three applications of Headline, at mid bloom, late bloom, and 10 days after flowering. Disease and agronomic data were collected and statistically analyzed following the same methods as for the earlier field trial.

## Results

### Phenotypic evaluation of the MDP

Median response scores resulting from inoculations with race 73 of *C. lindemuthianum* ranged from 1 (highly resistant) to 3 (highly susceptible) for the 266 MDP genotypes inoculated at the trifoliate leaf stage. A total of 236 genotypes and the susceptible checks Eclipse and Stampede displayed a susceptible reaction (median >1.0). Thirty genotypes displayed resistance (median = 1.0) (**Table 1**), including the resistant checks Envoy and USPT-ANT-1. Any previously reported anthracnose resistance gene for each of these 30 genotypes is listed on **Table 1**. Of the 30 genotypes, 6 genotypes were blacks and only UI-911 does not have a known anthracnose resistance gene. CDC Nordic was the only resistant great northern genotype and contains *Co-1^5^* (Dongfang et al., 2008). All seven resistant navy genotypes contain known resistance genes. One navy genotype, Huron, has been reported as partially resistant though it contains *Co-1* which should confer full resistance to race 73. Only one pink genotype, I9365-25, demonstrated resistance with no known resistance genes. Ten pinto genotypes exhibited resistance with seven having no known resistance genes. The PVP certificate indicates GTS-900 does demonstrate resistance to race 23 (delta), race 17 (alpha), race 130 (beta), and race 102 (gamma) but no resistance to race 73 has been reported. The remaining five genotypes were red or small red and contained no known resistance genes. Sixteen of the genotypes have at least one known anthracnose resistance gene and have been used in cultivar development. The remaining 14 genotypes with unknown resistance genes plus GTS-900 and Huron underwent further greenhouse testing to confirm their resistance and additionally characterize the resistance in these 16 genotypes with both unifoliate and trifoliate inoculations (**Table 2**).

**Table 2:** The disease reaction of 16 genotypes with observed ARR to race 73 reevaluated under controlled environmental conditions and various growth stages.

		Age inoculated/number of days in humid environment			
Reaction Category^a^	Genotype	Unifoliate/3-day	Trifoliate/5-day	Trifoliate/7-day	Trifoliate/9-day
Resistant Check	Envoy	1.2	0.9	2.5	0.8
Resistant Check	USPT-ANT-1	1.1	1.7	2.9	2.0
A	Dehoro	1.0	0.5	2.9	0.8
A	UI-911	1.1	0.5	2.1	2.6
B	GTS-900	1.5	2.5	3.9	8.2
B	Huron	1.8	1.3	2.7	8.0
C	Agassiz	1.7	6.7	8.7	5.0
C	CDC Pintium	2.3	6.7	6.9	8.2
C	CDC WM-2	3.0	7.5	8.7	8.2
C	Grand Mesa	2.2	6.8	8.7	5.6
C	Remington	2.6	7.1	7.3	5.2
C	Topaz	2.0	7.5	8.1	7.6
D	CENTA Pipil	7.3	2.7	3.7	8.2
D	F07-014-22-2	7.5	2.9	5.5	8.2
D	Merlot	7.1	4.5	3.4	8.2
E	F07-449-9-3	7.7	6.7	7.4	8.2
E	I9365-25	7.7	7.1	6.7	8.2
E	Maverick	7.2	7.7	6.1	8.0
Susceptible Check	Eclipse	7.3	7.7	9.5	8.2
Susceptible Check	Stampede	7.9	7.5	8.0	8.0

a Lines were grouped by disease reactions across all tests. A indicates resistance at all screening stages and conditions. B indicates resistance under unifoliate and trifoliate screening except under the 9-day incubation. C indicates resistance under unifoliate screening only. D indicates susceptibility under unifoliate screening and resistance or moderate resistance at the trifoliate 5-day or 7-day incubation. E indicates a susceptibility at all screening stages and conditions. Reactions were evaluated with a traditional 1-9 scale and adjusted using LSMeans.

### GWAS

**Figure 1:**
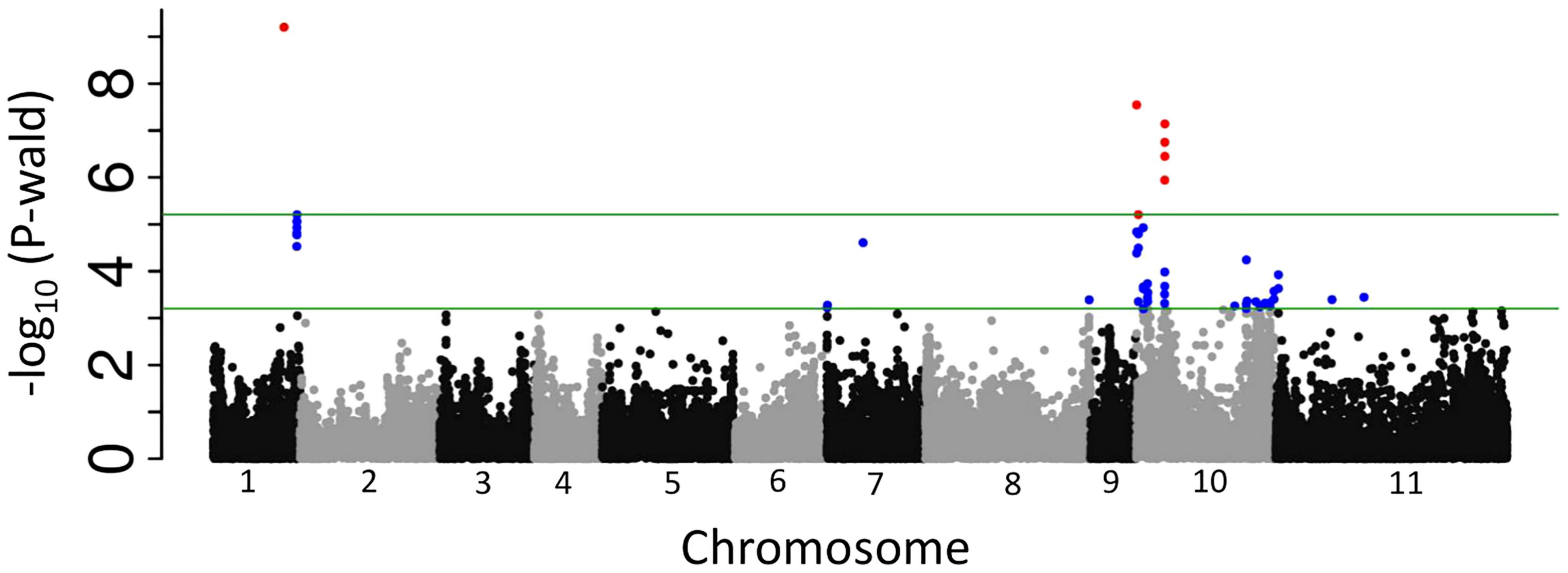
Manhattan plot of anthracnose resistance to race 73. The upper horizontal bar represents the cutoff for the most 0.01% significant single-nucleotide polymorphisms (SNP) (depicted in red). The lower horizontal bar represents the cutoff for the 0.1% significant SNP (depicted in blue). Both cutoffs are based on 1,000 bootstraps of the empirical distribution of the p-values.

A GWAS carried out with the MDP genotypes identified four physical regions associated with anthracnose resistance (**Figure 1**) and explained 26% of the observed phenotypic variation (**Table 3**). The peak SNP on Pv10, S10_72250, was responsible for most of the observed variation (23.4%) with the other 3 regions contributing 1% or less to the accumulative variation. The physical region as delineated by changes in minor allele frequency encompassed the region of 50kb to 80kb on Pv10 in which only one gene model was identified in this interval, *PvUI111.10G000300* with two splice variants. It encodes a ZPR1-like zinc-finger domain protein in which the peak SNP was located within intron 6. The other region on Pv10 (8.61-8.74 Mb) was represented by peak SNP, S10_8708314 and is near the location reported by Vidigal Filho et al. (2020). Several gene models associated with disease resistance are located within this region including *PvUI111.10G052000* encoding a cytochrome P450 51G1 protein and *PvUI111.10G052500* encoding a vacuolar sorting protein. The remaining two regions were both on Pv01 and coincided with regions previously associated with anthracnose resistance and individually contribute 1% or less to the total phenotypic variation. The first region, located at 48.41-48.51 Mb, encodes multiple genes including one for a cytochrome P450 polypeptide (*PvUI111.01G144600*) and multiple genes encoding NADH-ubiquinone/plastoquinone oxidoreductase chains. The last region on Pv01 (59.97-60.16 Mb) encodes fifteen different proteins including a CRINKLY4-related protein (PvUI111.01G254100). This gene was previously identified as anthracnose resistance gene *Co-x* by comparative genomics of a diversity panel and expression experiments (Richard et al., 2014; Richard et al., 2021).

**Table 3:** Chromosome, base pair position, p-value, and variation of each of the four peak SNP using the UI111 v1.1 location.

Location			Peak SNP			
Chromosome	Genomic Region	Peak SNP Position	Comparable SNP Location (G19833)	-log10 p	Variation	Cumulative Variation
	(Mb)	(Mb)			(%)	(%)
Pv01	48.41-48.51	S01_48462800	S02_44783121	9.20	0.2	26.5
Pv01	59.97-60.16	S01_60066578	S01_49585443	5.21	1.0	
Pv10	0.05-0.08	S10_72250	scaffold 73	7.55	23.4	
Pv10	8.61-8.74	S10_8708314	S10_8246946	9.20	0.2	

### Prolonged favorable disease environment evaluation on selected genotypes

All 16 resistant MDP genotypes with no known anthracnose resistance genes, plus the resistant and susceptible checks, underwent additional greenhouse testing to confirm their earlier identified resistance and further characterize the resistance. The first experiment consisted of inoculation of the unifoliate and three days of incubation to replicate traditional methods for anthracnose screening. Of the 16 genotypes tested, 10 were resistant and 6 were susceptible (**Table 2**). The second experiment, trifoliate inoculation and five days incubation, produced seven resistant genotypes, however all seven were not the same genotypes resistant during the unifoliate screening as seen on **Table 2**. The third experiment, trifoliate inoculation with seven days incubation, had six genotypes exhibiting resistance with F07-014-22-2 exhibiting a breakdown in resistance when compared to only 5 days of incubation. The fourth experiment, trifoliate inoculation with nine days incubation, saw the breakdown of resistance in all but two lines (**Table 2**). In the fifth experiment, all genotypes displayed a susceptible reaction after twelve days of incubation under humid conditions and thus the results were excluded from further analysis and tables.

Overall, the sixteen genotypes could be broken into five groups based on their reactions across all the experiments (**Table 2**). Group A contains both Dehoro and UI-911 which have a resistance level similar to the positive controls of USPT-ANT-1 and Envoy with similar levels of resistance in all four experiments. In group B the resistance observed in GTS-900 and Huron broke down under the longer 9-day incubation period. The next group of genotypes, C, exhibited resistance after unifoliate inoculation but were only partially resistant or susceptible in all the trifoliate inoculation experiments and they included Agassiz, Topaz, Grand Mesa, CDC Pintium, Remington, and CDC WM-2. Group D consisted of Merlot, CENTA Pipil, and F07-014-22-2. Those genotypes exhibited susceptibility during the unifoliate inoculation test but displayed resistance during the shorter incubations of the trifoliate inoculation tests. The last group, E, consisted of Maverick, F07-449-9-3 and I9365-25 and those lines were susceptible in each of the experiments.

### Field testing of partial resistance and fungicide spray regimes

USPT-ANT-1, GTS-900, Remington, Merlot, Maverick and Stampede, genotypes from each of the resistance reaction categories from **Table 2**, were field tested for anthracnose resistance. In the first field experiment, the two treatments were no anthracnose inoculation and supplemental anthracnose inoculation. Treatment had no significant effect on seed size or yield (**Supplementary Table 1**). No significant differences were observed between treatments for USPT-ANT-1 (resistant check), GTS-900, and Remington for canopy disease severity, pod disease severity, or seed health as there was little disease development in either treatment in any of the three years. Significant treatment differences were identified for Maverick in pod disease severity (2019: 22.3% vs 0.2%) and seed health (2019: 43.7 vs 1.7 ratio) but not for canopy disease severity. Stampede (susceptible check) had significant treatment differences in pod disease severity (2019: 32.3% vs 0.8%) and seed health (2019: 54.0 vs 1.7 ratio) but not in canopy disease severity. Merlot had significant treatment differences in canopy disease severity (2019: 11.7% vs 0.0%), pod disease severity (2019: 28.7% vs 1.1%) and seed health (2019: 66.7 vs 9.7 ratio).

The second field experiment had two treatments as well, the first was one application of fungicide during mid-bloom and the second was a series of three fungicide applications beginning mid-bloom. The fungicide application regime had no significant difference on canopy disease severity, seed weight, or yield for any of the genotypes (**Supplementary Table 2**). No significant differences were observed for Stampede (susceptible check), Merlot or Maverick in pod disease severity or seed health in 2017 or 2018. The conditions in 2019 were more favorable to disease development midseason and thus significant treatment differences were identified for Maverick, Merlot, and Stampede (susceptible check) in pod disease severity and seed health. However, Maverick and Merlot had similar levels of disease compared to the susceptible check, indicating a reduction in fungicide application is not appropriate to reduce anthracnose spread in ARR genotypes that are susceptible at the seedling stage.

## Discussion

The phenotyping of older plants (trifoliate stage) during the initial round of MDP screening focused on the identification of genomic regions contributing to ARR which could be targeted for use in pyramiding disease resistance. The standardized unifoliate screening method would not allow detection of ARR as infection in this method occurs during unifoliate leaf expansion. Previous research in dry beans indicated a major developmental shift occurs between unifoliate leaf expansion and trifoliate leaf expansion (Bateman and Lumsden, 1965; Beebe and Pastor-Corrales, 1991; Voysest, 2000; Bigirimana and Höfte, 2001) and thus was the focus of this research. Another major developmental shift occurs when plants change from a vegetative to reproductive stage which was not evaluated in this study. The use of a condensed scale to score the MDP limited the comparison of the results to previously published research. However, the differences in disease rating scales gave a more robust picture of disease reaction. The 1-3 disease severity rating considered the general appearance of the plant and produced better ratings for surviving/recovering plants after a five day or longer exposure time under favorable disease conditions.

The SNP markers associated with anthracnose ARR were located on Pv10 and on Pv01 with the primary factor near the end of Pv10. Major anthracnose resistance genes have been identified on Pv01 but not on Pv10. López et al. (2003) did mention an insignificant anthracnose resistance locus on Pv10 for race 7 but no physical location was identified. Zuiderveen et al. (2016) also identified an insignificant anthracnose resistance locus on Pv10 for race 7 near 3.78 Mb (G19833 v1.0). The identification of the ARR associated SNP, S10_72250 near the ZPR1 zinc protein finger protein is highly indicative of its involvement in anthracnose ARR. The ZPR1 protein is primarily localized in the cytoplasm but can accumulate in the nucleus when the cell is activated with mitogens. ZPR1 is an essential protein critical for normal cellular proliferation; however, its specific role is not known but it is hypothesized to function during regulation of pre-ribosomal RNA expression. ZPR1 interacts with the cytoplasmic domain of the inactive EF1A (elongation growth factor 1A) receptor and may inhibit its tyrosine kinase activity. The traditional role of EF1A is the delivery of aminoacyl-tRNA complex to ribosomes for protein elongation; however, EF1A is a multifunctional protein (Sasikumar et al., 2012; Momčilović and Fu, 2014). It is involved in the export of charged tRNA from the nucleus, arrangement of filamentous actin, as well as chaperone activity. As a chaperone it prevents thermal aggregation of malate dehydrogenase and may have been involved in salt tolerance in Arabidopsis (Shin et al., 2009). EF1A is highly expressed in young developing tissue but expression changes during plant development and in various tissues (Carneiro et al., 1999; Xu et al., 2007; Ransom-Hodgkins, 2009). EF1A expression is affected by environmental stimuli. In rice, expression increased after exposure to cold, drought, salt, or heat (Li and Chen, 1998). In barley (*Hordeum vulgare* L.) and maize (*Zea mays* L.), EF1A expression increased after exposure to cold stress (Dunn et al., 1993; Berberich et al., 1995). In soybeans, exposure to drought, cold, salt or ABA increased expression (Chung et al., 2009). In another system, various isoforms of EF1A were upregulated during the hypersensitive response (HR) after induction in grapes (*Vitis vinifera* Linnaeus) (Dietrich et al, 2010). The reported differences support the role of EF1A in reactions to stress and with its interaction with ZPR1, make ZPR1 a possible target for exploiting anthracnose ARR.

The other resistance regions identified in this study had a minor influence on anthracnose ARR in this study, each explaining less than 1% of the phenotypic variation. The interval containing the peak SNP, S01_48462800, encoded several proteins with a known role in disease resistance including Cytochrome P450 (reviewed by Pandian et al., 2020) and may be the same resistance loci identified by Zuiderveen et al. (2016) and fine mapped by Murube et al. (2019). *Co-1* resides on Pv01 and may fall within this interval. The interval containing the peak SNP S01_60066578 included a CRINKLY4 related protein. Recently Richard et al. (2021) identified a truncated CRINKLY4 kinase as a candidate for *Co-X*, corroborating our results.

**Figure 2:**
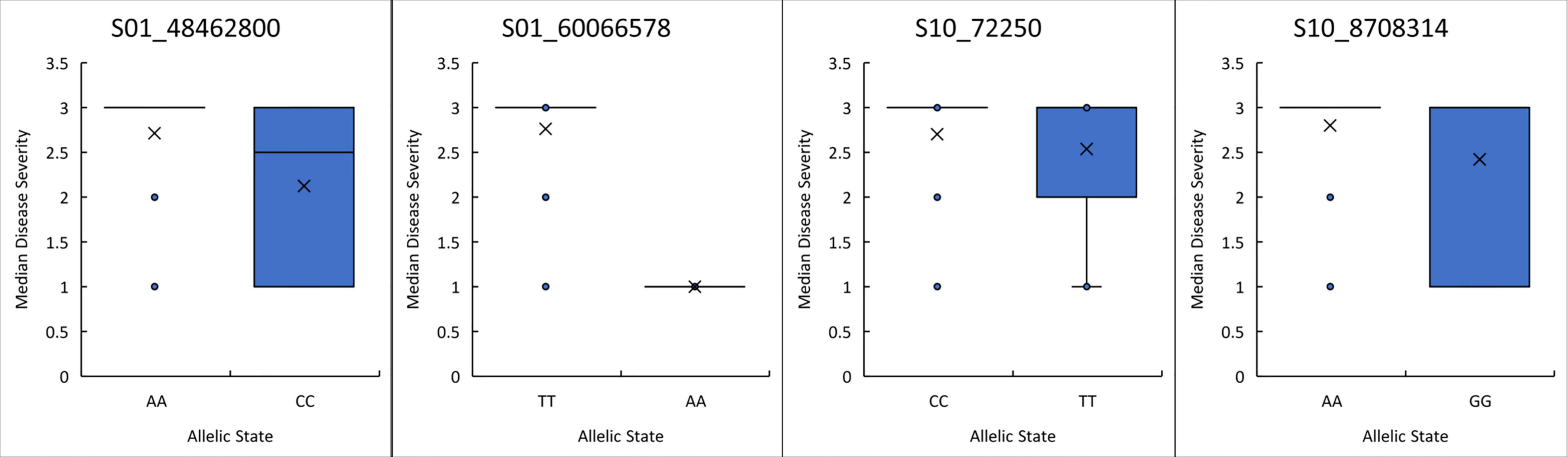
Boxplot of anthracnose resistance scores for the allelic state of each of the peak SNPs identified on Pv01 and Pv10. The student’s t-test indicated the allelic state means were significantly different for S01_48462800, S01_60066578, and S10_8708314 with probabilities of 0.01, 0.00 and 0.00, respectively.

The predictive utility of each of the four peak significant SNPs was evaluated by comparing the means between the two allelic states for each peak SNP (**Figure 2**). Three of the four peak SNPs, S01_48462800, S01_60066578, and S10_8708314, had significantly different means for each allelic state using a student’s t-test (0.012 0.00, and 0.00 probability respectively). A closer look at the phenotypes for each allelic state indicates SNP S01_60066578 may be a good predictor for breeding as all genotypes with an adenine at position S01_6006578 exhibited a resistant reaction. This is consistent with the expected dominant mode of inheritance for most anthracnose resistance genes reported. SNPs, S01_48462800 and S10_8708314, are not as good a predictor as S01_6006578 since the phenotypes were not all resistant. These two SNPs may not be as close to the genes regulating the race 73 resistance response in their respective locations, or perhaps the mechanism behind the resistance is not simple dominance. The disease means are not significantly different for S10_72250 and thus it would be difficult to use it to predict a resistant phenotype. As stated by Lo et al. (2015) significance analyses are made based on assumptions of the underlying distribution and predictive analyses are based on knowledge of the underlying distribution. Therefore, variables that are highly significant may not be highly predictive. Further study is required to understand the mechanism underlying resistance at this location.

Differences in the Andean reference genome G19833 and the Middle American genome UI111 suggests careful thought should be used when selecting a reference genome for each study. Many SNP datasets are being generated without using the scaffold sequences. This study focused on genotypes in the Middle American gene pool and thus a reference genome genotype in the Middle American gene pool was selected. The use of the Andean reference would have eliminated S10_72250 as it was on scaffold 73 (**Table 3**). A variation in the reference genome assemblies place SNP S01_48462800 on chromosome 1 in UI111 but on chromosome 2 in G19833. The association results would have been different removing one highly significant SNP and shifting another highly significant SNP to another chromosome. Structural differences between the reference genomes and the lack of phenotypic data make it difficult to compare underlying genes and their expression. With the availability of multiple reference genomes in dry bean, careful evaluation of the genotypes used in the study should be undertaken to select the most appropriate reference genome.

Most known anthracnose resistance genes to race 73 derive either from Andean genotypes, with the multiallelic *Co-1* resistance gene family, such as A193 with *Co-1* (Mendoza et al., 2001), Kaboon with *Co-1^2^* (White Kidney; Zuiderveen et al, 2016), and Widusa with *Co-1^5^* (Gonçalves-Vidigal and Kelly, 2006). The *Co-4^2^* gene, derived from the Andean genotype SEL1308 (Alzate-Marin et al., 1997), is also known to provide anthracnose resistance to race 73. Middle American genotypes, with genes from the *Co-3* resistance gene cluster derived from the genotypes Mexico 222 (White; *Co-3*; Méndez-Vigo et al., 2005; Rodríguez-Suárez et al., 2008), BAT93 (Tan; *Co-9*; Geffroy et al., 1999) and Ouro Negro (black; *Co-10*; Alzate-Marin et al., 2003) provide anthracnose resistance to several anthracnose races, however, from the *Co-3* resistance gene cluster only *Co-10* provides resistance to race 73. All these resistance genes provide protection against anthracnose infection from early stages onwards and are not known to be age-related. The results from this research study suggest that some resistance to anthracnose may be ARR, and that breeders can target ARR for pyramiding resistance.

The extended greenhouse testing confirmed the presence of anthracnose resistance or intermediate resistance in 13 of the 16 MDP lines and further characterized their resistant behavior. The 1-9 scale used in the extended greenhouse testing is more comparable to other studies and facilitated the identification of the type of ARR. The additional exposure times of disease pressure also showed some genotypic variation across time for the genotypes and allowed the genotypes to be further classified as highly resistant, highly resistant with abrupt resistance breakdown, only early resistance, early susceptibility progressing to short-lived resistance, or a susceptible rating (**Table 2**). Resistance was not confirmed in either Maverick or F07-499-9-3. Both genotypes were near the breakpoint between resistant and susceptible using the condensed 1-3 scale during the trifoliate screening. Both genotypes had multiple plants with no symptoms but also some plants with symptoms and healthy regrowth tissue. Several reasons can explain the variation. It is possible the variation was due to escapes. Based on the number of plants that would have been escapes in just these two particular lines, it’s unlikely the mixed phenotype is due to escapes. A second possible explanation is these two genotypes were not homozygous for resistance. One example of this type of variation in dry beans was rust resistance in ‘Stampede’. Stampede was released as a genotype resistant to rust with multiple genes for rust resistance. A new race of rust appeared and ‘Stampede’ was found to segregate for resistance. The resistance gene for the new race of rust was not under any selection pressure and allelic frequency had reached nearly 50:50. A third explanation is the seed source was not pure. A fourth possibility is the difference in statical measure usage. Use of the medians in the initial trifoliate screening eliminated the outliers but would not account for segregation. However, segregation would affect the LSMeans in the extended testing. Another possibility is the presence of a mechanism which slows disease progression and suggests partial or quantitative resistance. Understanding the various resistance mechanisms will allow breeders to further utilize that knowledge for cultivar development.

Field testing confirmed the resistance observed in GTS-900 and Remington and by extension suggests Huron, Agassiz, Topaz, Grand Mesa, CDC Pintium, and CDC WM-2 would exhibit high levels of anthracnose resistance under field conditions, but this remains to be confirmed. The field-testing results did not mirror the greenhouse results. In the greenhouse, plants were inoculated at two different stages, the unifoliate (10 days after planting) and first trifoliate (20 days after planting). The field inoculum was applied later (~35 days after planting). The expectation was the trifoliate reaction in the greenhouse would more closely match the field reaction; however, the greenhouse unifoliate reaction was a better indicator of how the genotypes performed in the field. Typically, the unifoliate reaction detects the presence of R genes that tend to be pathogen and race specific. These genes tend to have a large effect and were more likely the ones observed in the field. The ARR genes tend to be “resistance boosters” or result in broad spectrum partial resistance, neither of which are easily observed under field conditions. The environmental conditions in the greenhouse were tightly controlled, thus generating a more specific picture of the resistance reaction.

In this study, MDP genotypes which exhibited ARR to anthracnose race 73 were mostly from the pinto market class of beans (i.e., Agassiz, CDC Pintium, CDCWM-2, Grand Mesa, GTS-900, Remington, and Topaz). However, the navy bean, Huron, also expressed ARR. Three red genotypes exhibited ARR to anthracnose: CENTA Pipil (small red), F07-014-22-2 (red) and Merlot (small red). Two genotypes, Deorho (small red) and UI-911 (black) were fully resistant compared to the resistant checks. The benefits of the newly identified resistant genotypes include reductions in fungicide spray regimes, minimizing the environmental impact, as well as decreasing losses in seed yield and quality, all results that provide an economic benefit to the growers. Further tests are needed to demonstrate the extent of these benefits. Certainly, these genotypes will be valuable new sources for anthracnose resistance for breeding programs. Two clusters of resistance genes on Pv01 and Pv10 were shown to be associated with a resistant phenotype to race 73 of *C. lindemuthianum* within a region on Pv10 containing the primary gene for ARR resistance. However, only anthracnose race 73 has been tested so far and it remains to be seen if the genes in these genomic regions can lead to durable resistance or tolerance to other races.

## Conclusion

With losses of up to 100% seed yield, anthracnose is a devastating disease on dry beans and resistance is usually controlled by major genes clustered in several genomic regions. Typically, seedlings are screened for anthracnose resistance and resistance that develops during later vegetative growth stages is missed. A Genome-Wide Association Study for anthracnose age-related resistance identified two chromosomes containing race 73 resistance using a Middle American diversity panel. Two identified genomic regions were described previously on Pv01, likely corresponding to the multiallelic *Co-1* loci and *Co-X*. Two new regions were identified on Pv10, one of which contains a *ZPR1*-like gene and is responsible for 23% of the observed variation. The SNPs identified in this new genomic region will be validated and targeted for future marker development to combine multiple anthracnose resistance genes with different modes of action into a single cultivar. The anthracnose disease testing results carried out in the greenhouse were validated using representative cultivars in the field. Field testing confirmed anthracnose resistance to race 73 in two pinto bean cultivars (GTS-900 and Remington). Field testing also confirmed fewer applications of fungicide were effective in preventing significant differences in anthracnose development when these resistant cultivars were used.

## Data availability statement

The datasets presented in this study can be found in online repositories. The names of the repository/repositories and accession number(s) can be found below: http://arsftfbean.uprm.edu/beancap/research/, HapMap_469-Pinto UI111-geno-GATK-cleaned-imputed-sorted-hpmp-hmp, version: 1.0.

## Author contributions

SS, JO and RC conceived the experiments. SS carried out the greenhouse phenotyping on the MDP. WP, DS and RC completed the field testing, initial statistical analysis on field results, and confirmed greenhouse phenotyping on the field-tested lines. AO and PM contributed the MDP SNP dataset. KS completed the GWAS conducted the final statistical analysis and wrote the manuscript. All authors contributed to the article and approved the submitted version.

## Funding

This project was supported by the United States Department of Agriculture – Agricultural Marketing Service (USDA-AMS) and the North Dakota Department of Agriculture through the Specialty Crop Research Block Grant Program (16-SCBGP-ND-0029). Its contents are solely the responsibility of the authors and do not necessarily represent the official views of the USDA. Additional partial support came from the Northarvest Bean Growers Association and Agriculture and Agri-Food Canada. This work used resources of the Center for Computationally Assisted Science and Technology (CCAST) at North Dakota State University, which were made possible in part by NSF MRI Award No. 2019077. 

## Acknowledgments

Thank you to the NDSU Pulse Pathology Program for providing *C. lindemuthianum* race 73 for greenhouse inoculations. 

## Conflict of interest

The authors declare that the research was conducted in the absence of any commercial or financial relationships that could be construed as a potential conflict of interest.

## Publisher’s note

All claims expressed in this article are solely those of the authors and do not necessarily represent those of their affiliated organizations, or those of the publisher, the editors and the reviewers. Any product that may be evaluated in this article, or claim that may be made by its manufacturer, is not guaranteed or endorsed by the publisher.
